# Melatonergic Modulation of SIRT1-Nrf2 Signaling Protects Against Doxorubicin-Induced Hepatic Injury in Rats

**DOI:** 10.3390/biomedicines14071468

**Published:** 2026-06-28

**Authors:** Haluk Kerim Karakullukcu, Hatice Aygun, Murat Kalın, Mina Karakullukcu, Aylin Arslan, Serdar Savaş Gül, Ömer Faruk Özkan, Gülçin Ercan

**Affiliations:** 1 Department of General Surgery, Sultan 2. Abdulhamid Han Educational and Research Hospital, University of Health Sciences, Istanbul 34668, Türkiye; halukkarakullukcu@gmail.com (H.K.K.); murat.kalin@hotmail.com (M.K.); omerfaruk.ozkan@sbu.edu.tr (Ö.F.Ö.); ghepgul@hotmail.com (G.E.); 2 Neuroscience Laboratory, BAMER, Biruni University, Istanbul 34015, Türkiye; 3 Department of Oncology, Sultan 2. Abdulhamid Han Educational and Research Hospital, University of Health Sciences, Istanbul 34668, Türkiye; mina.zoralioglu@gmail.com; 4 Department of General Surgery, Istanbul Prof. Dr. Cemil Taşçıoğlu City Hospital, University of Health Sciences, Istanbul 34668, Türkiye; aylinarslan53@outlook.com; 5 Department of Nuclear Medicine, Faculty of Medicine, Lokman Hekim University, Ankara 06530, Türkiye; serdar.gul@lokmanhekim.edu.tr

**Keywords:** doxorubicin, hepatotoxicity, melatonin, agomelatine, oxidative stress, SIRT1, scintigraphic imaging

## Abstract

**Objectives:** Doxorubicin (DOX) is an effective chemotherapeutic agent, but its clinical use is limited by hepatotoxicity associated with oxidative stress and inflammatory signaling. This study aimed to investigate the potential protective effects of melatonin and agomelatine on DOX-induced hepatic injury using scintigraphic, biochemical, and molecular parameters. **Methods:** Twenty-eight rats were randomly divided into four groups: Control, DOX, DOX + Melatonin, and DOX + Agomelatine. Melatonin and agomelatine were administered as a one-week pretreatment, and DOX was injected during the experimental period (total dose: 18 mg/kg). Hepatic injury was evaluated using 99mTc-PYP scintigraphic imaging, serum liver enzymes (AST, ALT, and LDH), oxidative stress markers (MDA, GSH, Nrf2, and SIRT1), and inflammatory cytokines (TNF-α, IL-6, and IL-10). **Results:** DOX administration significantly increased hepatic 99mTc-PYP uptake, as well as serum AST, ALT, and LDH levels, indicating hepatocellular necrosis and membrane damage. DOX also increased MDA, TNF-α, and IL-6 levels while reducing GSH, Nrf2, SIRT1, and IL-10, demonstrating pronounced oxidative stress and inflammatory activation. Treatment with melatonin or agomelatine significantly reduced tracer uptake and liver enzyme levels compared with the DOX group. Both treatments improved antioxidant status by decreasing MDA and restoring GSH, Nrf2, and SIRT1 levels, while simultaneously attenuating inflammatory cytokine responses through reduction in TNF-α and IL-6 and partial restoration of IL-10. **Conclusions:** Melatonin and agomelatine attenuated DOX-induced hepatotoxicity by reducing oxidative stress, modulating inflammatory responses, and restoring hepatic SIRT1 and Nrf2 levels. These findings suggest that melatonergic interventions may represent promising protective strategies against doxorubicin-induced liver injury.

## 1. Introduction

Doxorubicin is an anthracycline with potent antineoplastic efficacy against a wide range of solid and hematological malignancies; however, owing to its extensive hepatic biotransformation and biliary elimination, it exhibits a biologically vulnerable pharmacokinetic profile with respect to hepatotoxicity [1,2,3]. Although elevations in serum aminotransferases are often transient and asymptomatic in clinical practice, both experimental and clinical evidence indicate that oxidative stress, mitochondrial dysfunction, lipid peroxidation, and inflammatory cytokine activation constitute the principal determinants of doxorubicin-induced liver injury [4,5]. Reactive oxygen species generated during the redox cycling of doxorubicin not only deplete endogenous antioxidant reserves, such as glutathione, but also induce structural damage to membrane lipids and mitochondrial proteins, thereby compromising hepatocyte integrity [5,6,7]. Concurrently, increased levels of pro-inflammatory mediators, including TNF-α and IL-6, together with reduced anti-inflammatory signaling such as IL-10 and activation of cell death pathways, suggest that doxorubicin hepatotoxicity reflects not merely an increase in oxidative burden but rather a complex disruption of the redox–inflammation axis [6,8,9].

Consequently, contemporary hepatoprotective strategies have shifted toward multi-targeted approaches that simultaneously modulate iron-dependent oxidative injury, transcriptional antioxidant responses, inflammatory signaling networks, and mitochondrial homeostasis rather than focusing on a single downstream pathway [5,10]. Although dexrazoxane is the most extensively characterized anthracycline-protective agent in clinical practice, its use has been primarily established for cardioprotection, and robust evidence supporting its hepatoprotective efficacy remains limited, underscoring the need for novel liver-directed adjuvant therapies [1,11]. At the experimental level, Nrf2 activators and compounds that enhance the Keap1/Nrf2/HO-1 signaling axis have been shown to attenuate doxorubicin-induced hepatocellular injury by reducing oxidative stress, whereas agents such as omega-3 fatty acids, pregnenolone, and ginkgetin have demonstrated the ability to reinforce antioxidant defenses while suppressing inflammatory and apoptotic mediators [7,12,13]. Similarly, modulation of SIRT1 has emerged as a promising hepatoprotective strategy because SIRT1 orchestrates multiple cellular defense mechanisms associated with energy metabolism, mitochondrial biogenesis, NF-κB suppression, and antioxidant gene expression [14,15]. Among natural antioxidants and anti-inflammatory compounds, diosmin, luteolin, naringenin, and various plant-derived flavonoids have produced encouraging results through reductions in malondialdehyde levels, restoration of glutathione reserves, suppression of inflammatory cytokines, and attenuation of apoptotic signaling pathways; nevertheless, many of these compounds remain at the stage of experimental validation [6,8,9]. Mitochondrial protective strategies have also gained increasing attention because doxorubicin-induced calcium dysregulation, disruption of the electron transport chain, and loss of mitochondrial membrane potential facilitate both necrotic and apoptotic cell death [5,15,16]. Collectively, these findings suggest that the most effective candidates for preventing doxorubicin-induced hepatotoxicity are those capable of simultaneously suppressing oxidative stress and inflammation while activating endogenous cellular resilience programs [7,10,15].

Within this context, melatonin remains one of the most compelling biological candidates for experimental hepatoprotection because it directly scavenges free radicals, supports glutathione recycling, limits mitochondrial permeability transition, and enhances Nrf2-dependent antioxidant gene programs [11,17]. In models of liver injury, melatonin has consistently been shown to reduce lipid peroxidation, attenuate aminotransferase elevations, and shift cytokine profiles toward a more anti-inflammatory phenotype, indicating that this molecule functions not only as an antioxidant but also as an immunometabolic modulator [17,18,19]. In contrast, agomelatine mimics melatonergic signaling via MT1/MT2 receptor agonism and additionally exerts 5-HT2C receptor antagonism, thereby influencing neuroendocrine and circadian regulation through a distinct pharmacological profile. This characteristic may render agomelatine particularly attractive as a dual-purpose adjunctive agent in oncology patients who frequently experience circadian disruption, anxiety, and depressive symptoms [20,21,22]. Nevertheless, the requirement for routine liver enzyme monitoring during agomelatine therapy necessitates careful interpretation of its potential hepatoprotective effects from a translational perspective [23,24]. Consequently, demonstrating a protective effect of agomelatine against doxorubicin-induced liver injury would represent a biologically paradoxical yet scientifically important finding, as a melatonergic modulator that is beneficial in acute oxidative–inflammatory injury models may behave through mechanisms distinct from the idiosyncratic hepatic events occasionally observed during chronic clinical use [23,25]. To our knowledge, direct comparative evaluation of melatonin and agomelatine in a doxorubicin-induced hepatotoxicity model remains extremely limited [17,24,25].

The current literature evaluating protective interventions against doxorubicin-induced hepatotoxicity has predominantly relied on histopathological assessments, serum liver enzymes, and conventional oxidative stress biomarkers. In contrast, the combined investigation of necrosis-sensitive functional imaging and molecular defense pathways within the same experimental paradigm remains remarkably limited [26,27,28]. Bone-avid radiotracers such as 99mTc-pyrophosphate have been shown to accumulate within calcium-rich necrotic tissues and have also been described in the setting of extensive hepatic necrosis, thereby providing a potential in vivo functional marker of hepatocellular membrane disruption and necrotic injury [26,27,29]. Simultaneous evaluation of SIRT1 and Nrf2, two key regulators of cellular antioxidant and inflammatory responses, offers the additional advantage of linking biochemical improvement to alterations in endogenous defense mechanisms [10,14,15].

Accordingly, the novelty of the present study lies in the comprehensive comparative assessment of melatonin and agomelatine against doxorubicin-induced hepatotoxicity using not only conventional serum biomarkers but also necrosis-sensitive scintigraphic imaging, oxidative stress parameters, inflammatory cytokine profiles, and hepatic SIRT1 and Nrf2 levels [10,25,26]. Such an integrated approach more accurately reflects the multifactorial nature of doxorubicin-associated liver injury and may contribute to a clearer understanding of the role of melatonergic modulation in experimental hepatoprotection [5,17,25]. Therefore, the present study investigated the potential protective effects of melatonin and agomelatine against doxorubicin-induced hepatotoxicity using scintigraphic, biochemical, and molecular assessments.

## 2. Materials and Methods

### 2.1. Animals

A total of forty-nine adult male Wistar rats weighing between 220 and 280 g were included in the study. The animals were obtained from the Experimental Research Center of Gaziosmanpasa University. All experimental procedures were conducted in accordance with institutional ethical guidelines and were approved by the Local Animal Ethics Committee of Gaziosmanpasa University (Approval No: 2017/15).

The rats were housed under controlled environmental conditions with a constant room temperature of approximately 23 °C, relative humidity of about 50%, and a 12 h light/dark cycle. Standard laboratory chow and tap water were provided ad libitum throughout the experimental period.

### 2.2. Experimental Design

**Figure 1:**
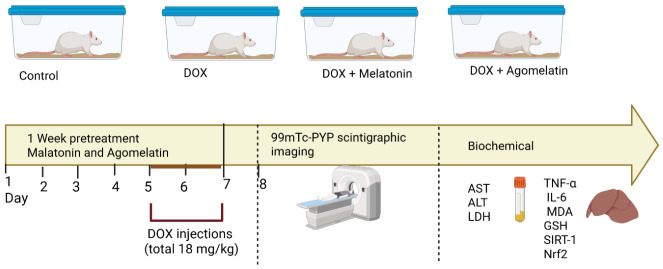
Experimental timeline.

The experimental protocol consisted of four groups, each including seven rats (n = 28). Animals were randomly assigned to the following groups (Figure 1).

Group I (Control): Rats in the control group received intraperitoneal saline (1 mL/kg body weight) once daily for seven consecutive days.

Group II (DOX): Animals in this group received doxorubicin to induce hepatic injury. Doxorubicin was administered intraperitoneally at a cumulative dose of 18 mg/kg for three consecutive days during the experimental period.

Group III (Melatonin + DOX): Rats were treated with melatonin (40 mg/kg, i.p.) once daily for seven days. In addition, doxorubicin was administered intraperitoneally at a cumulative dose of 18 mg/kg at 09:00 a.m. on days 5, 6, and 7.

Group IV (Agomelatine + DOX): Animals received agomelatine treatment (40 mg/kg, i.p.) once daily for seven consecutive days. As in the previous group, doxorubicin was administered intraperitoneally at a cumulative dose of 18 mg/kg on days 5, 6, and 7.

#### 2.2.1. Rationale for Dose Selection

The cumulative doxorubicin dose of 18 mg/kg administered over a short treatment period was selected because it is a widely used experimental approach for reliably inducing acute/subacute hepatotoxicity in rats, characterized by significant elevations in serum liver enzymes, oxidative stress, and inflammatory activation [7,25]. This dose range produces measurable biochemical and molecular injury while avoiding excessive mortality, thereby enabling adequate evaluation of the protective efficacy of experimental interventions [5,7,30].

The melatonin dose of 40 mg/kg was selected based on previous experimental studies demonstrating robust antioxidant, anti-inflammatory, and mitochondrial protective effects, particularly through suppression of lipid peroxidation and enhancement of GSH- and Nrf2-related antioxidant defenses [17,18,19]. Similarly, the agomelatine dose of 40 mg/kg was selected based on previous experimental studies demonstrating protective effects against oxidative–inflammatory injury and favorable modulation of melatonergic signaling pathways, including recent evidence in models of doxorubicin-induced hepatotoxicity [25].

Therefore, the combination of 18 mg/kg doxorubicin with 40 mg/kg melatonin or agomelatine was considered a rational experimental design capable of balancing a reproducible toxic stimulus with a measurable protective response within the same model [7,17,25].

#### 2.2.2. Rationale for Pretreatment Protocol

Administration of melatonin or agomelatine for 5–7 days prior to doxorubicin exposure was designed not only to ensure pharmacological availability at the time of injury induction but also to provide a form of biological preconditioning that may enhance endogenous cellular defense mechanisms [11,17]. This pretreatment period has been associated with activation of Nrf2-dependent antioxidant gene expression, partial restoration of intracellular glutathione reserves, improved mitochondrial stress tolerance, and attenuation of the pro-inflammatory priming that contributes to excessive NF-κB-mediated cytokine production [10,17,31].

In addition, prior enhancement of adaptive NAD+-dependent pathways, including SIRT1, may increase cellular resistance to the oxidative and inflammatory burden induced by doxorubicin administration, thereby supporting a mechanism-based rather than merely symptomatic protective strategy [11,14,15]. Accordingly, the one-week pretreatment schedule followed by doxorubicin administration on days 5–7 was selected as a biologically plausible and literature-supported protocol consistent with the principles of antioxidant preconditioning, SIRT1/Nrf2 priming, and mitochondrial adaptation [17,25,31].

### 2.3. Scintigraphic Imaging

For scintigraphic evaluation, rats were first anesthetized and subsequently injected intravenously with 1 mCi of technetium-99m pyrophosphate (99mTc-PYP). Static scintigraphic imaging was performed 60 min after tracer administration using a dual-head gamma camera system (E-CAM, Siemens, Erlangen, Germany). Images were acquired in both anterior and posterior projections at a magnification factor of 2.55.

To quantify radiotracer accumulation, regions of interest (ROIs) of identical rectangular size were manually drawn over the cardiac area in all images. Radiotracer uptake was then determined using a semi-quantitative ROI-based analysis, and the obtained uptake values were recorded for each experimental group.

### 2.4. Biochemical Assays and Measurement of Hepatic Enzymes

At the end of the experimental period, the animals were anesthetized, and blood samples were collected from each rat into sterile tubes. The collected blood samples were allowed to clot at room temperature for approximately 30 min and then centrifuged at 3000 rpm for 10 min to obtain serum. The separated serum fractions were carefully transferred into Eppendorf tubes and stored for subsequent biochemical analyses. Serum levels of aspartate aminotransferase (AST), alanine aminotransferase (ALT), and lactate dehydrogenase (LDH) were measured as indicators of hepatic injury.

Following blood collection, all animals were euthanized under deep anesthesia in accordance with ethical guidelines.

### 2.5. Tissue Preparation and Biochemical Analyses

All tissue samples were rinsed with cold isotonic saline solution (0.9% NaCl) to remove blood residues, and wet tissue weights were recorded. The tissues were then minced into small fragments using a sterile scalpel and transferred into homogenization tubes. Homogenization was performed at 4 °C in cold phosphate-buffered saline (PBS, pH 7.4) using stainless steel beads to obtain uniform tissue homogenates.

To obtain the supernatant fractions, the homogenized samples were centrifuged at 2500 rpm for 20 min, and the resulting supernatants were collected for subsequent biochemical analyses. Tissue levels of TNF-α, IL-6, IL-10, nitric oxide (NO), malondialdehyde (MDA), glutathione (GSH), SIRT-1, and NRF2 were determined using quantitative sandwich enzyme-linked immunosorbent assay (ELISA) kits according to the manufacturer’s instructions.

### 2.6. Statistical Analysis

All statistical analyses were performed using GraphPad Prism software (version 10, GraphPad Software, San Diego, CA, USA) and SPSS software (version 19, IBM Corp., Armonk, NY, USA). Data are expressed as mean ± standard error of the mean (SEM). The normality of the data distribution was assessed using the Shapiro–Wilk test. For normally distributed variables, differences among groups were analyzed using one-way analysis of variance (ANOVA) followed by Tukey’s multiple-comparison test. For variables that did not meet the normality assumption, the Kruskal–Wallis test followed by the Mann–Whitney U test was used. A *p*-value < 0.05 was considered statistically significant.

## 3. Results

### 3.1. Effects of Different Treatments on Hepatic Scintigraphic Uptake

**Figure 2:**
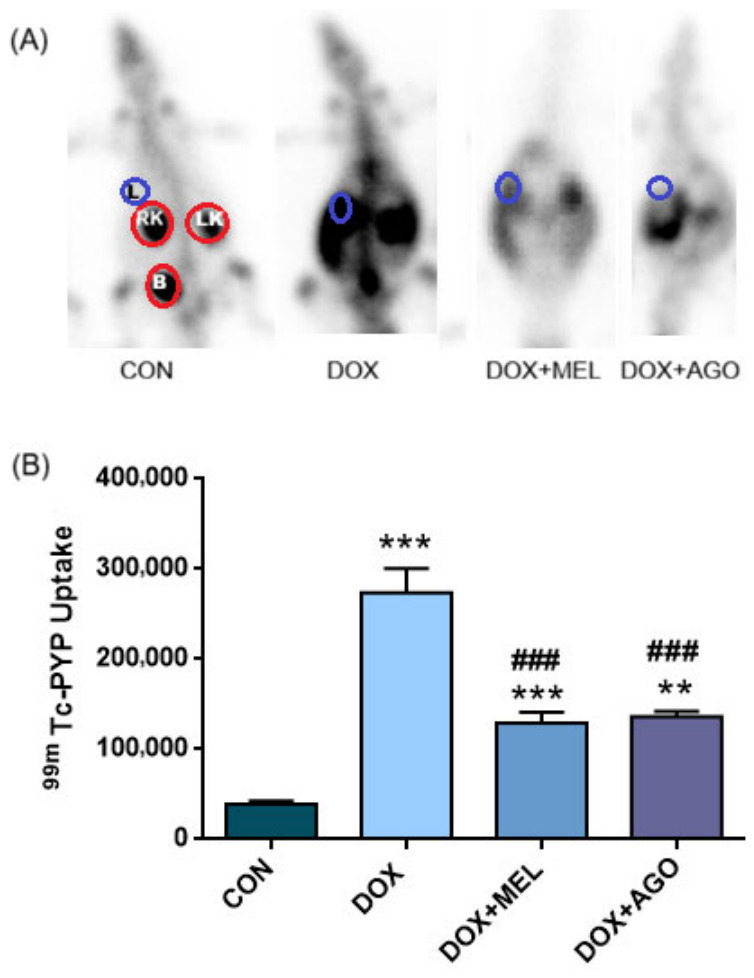
Representative 99mTc-pyrophosphate (99mTc-PYP) scintigraphic images and quantitative analysis of hepatic uptake: (**A**) Planar images showing tracer distribution in control, DOX, DOX + melatonin, and DOX + agomelatine groups. Regions corresponding to liver (L), right kidney (RK), left kidney (LK), and bladder (B) are indicated. Increased hepatic uptake is evident in the DOX group, while treatment groups show reduced tracer accumulation. (**B**) Quantitative analysis of hepatic 99mTc-PYP uptake. Data are presented as mean ± SEM. *** *p* < 0.001, ** *p* < 0.01 vs. control; ### *p* < 0.001 vs. DOX.

As illustrated in Figure 2 and Table 1, hepatic scintigraphic uptake differed significantly among the experimental groups. One-way ANOVA revealed a significant overall group effect (F(3,24) = 42.31, *p* < 0.0001). Compared with the control group, DOX administration significantly increased hepatic radiotracer uptake (*p* < 0.001), indicating severe hepatic injury. Co-administration of both melatonin and agomelatine significantly reduced hepatic scintigraphic uptake compared with the DOX group (*p* = 0.0014; *p* < 0.001, respectively). However, no statistically significant difference was observed between the DOX + melatonin and DOX + agomelatine groups (*p* = 0.9860) (Figure 2).

### 3.2. Effects of Different Treatments on Hepatic Injury Markers

**Figure 3:**
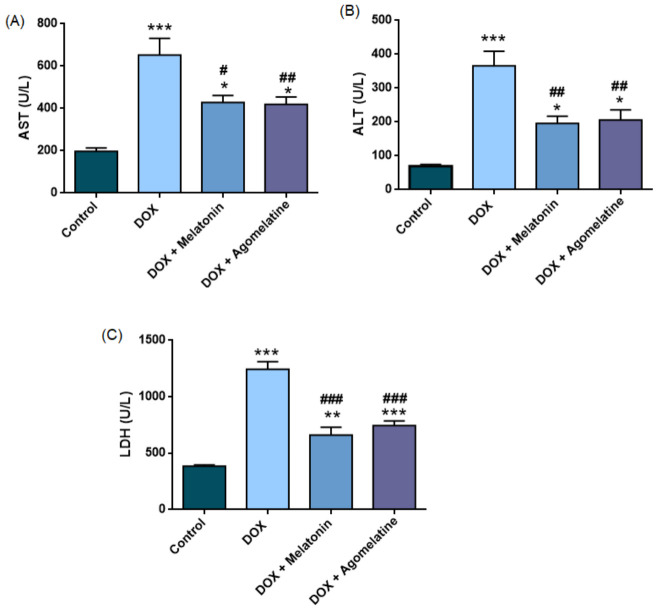
Effects of melatonin and agomelatine on DOX-induced hepatic injury markers: (**A**) AST, (**B**) ALT, and (**C**) LDH levels in the experimental groups. DOX administration markedly increased serum AST, ALT, and LDH levels compared with the control group, indicating severe hepatocellular injury. Treatment with melatonin or agomelatine significantly attenuated these DOX-induced increases. Values are expressed as mean ± SEM (n = 7 per group). Statistical analyses were performed using one-way ANOVA followed by Tukey’s multiple-comparison test. *** *p* < 0.001, ** *p* < 0.01, * *p* < 0.05 vs. Control; ### *p* < 0.001, ## *p* < 0.01, # *p* < 0.05 vs. DOX.

Hepatic injury markers differed significantly among the experimental groups, as shown in Figure 3 and Table 1. One-way ANOVA revealed significant overall group effects for AST (F(3,24) = 15.49, *p* < 0.0001), ALT (F(3,24) = 18.09, *p* < 0.0001), and LDH levels (F(3,24) = 44.96, *p* < 0.0001). Compared with the control group, DOX administration markedly increased serum AST, ALT, and LDH levels (*p* < 0.001 for all comparisons), indicating severe hepatocellular injury. Co-administration of both melatonin and agomelatine significantly attenuated DOX-induced hepatic injury, as evidenced by decreased serum AST (melatonin: *p* = 0.0130; agomelatine: *p* = 0.0095), ALT (melatonin: *p* = 0.0016; agomelatine: *p* = 0.0029), and LDH levels (*p* < 0.0001 for both treatments) compared with the DOX group. However, no statistically significant differences were observed between the DOX + melatonin and DOX + agomelatine groups for any of these parameters (*p* > 0.05) (Figure 3).

### 3.3. Effects of Different Treatments on Hepatic SIRT1 Levels

**Figure 4:**
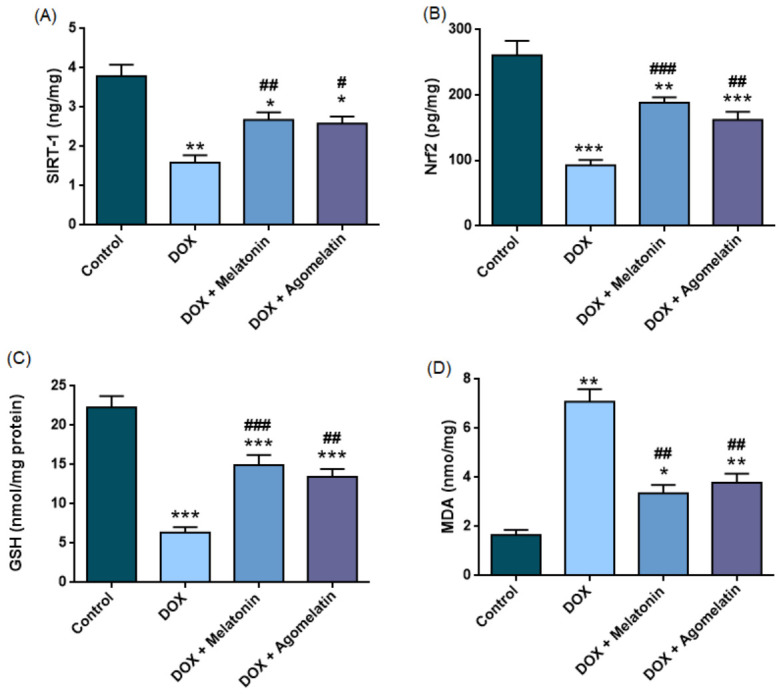
Effects of melatonin and agomelatine on hepatic oxidative stress markers in DOX-induced toxicity: (**A**) SIRT1, (**B**) NRF2, (**C**) GSH, and (**D**) MDA levels in liver tissue of the experimental groups. DOX administration significantly decreased SIRT1, NRF2, and GSH levels while significantly increasing MDA levels compared with the control group. Treatment with melatonin or agomelatine significantly restored SIRT1, NRF2, and GSH levels and attenuated the DOX-induced increase in MDA levels. Values are expressed as mean ± SEM (n = 7 per group). Statistical analyses were performed using one-way ANOVA followed by Tukey’s post hoc test for NRF2 and GSH and Kruskal–Wallis followed by Mann–Whitney U test for SIRT1 and MDA. * *p* < 0.05, ** *p* < 0.01, *** *p* < 0.001 vs. Control; # *p* < 0.05, ## *p* < 0.01, ### *p* < 0.001 vs. DOX.

As shown in Figure 4 and Table 2, hepatic SIRT1 levels differed significantly among the experimental groups. Kruskal–Wallis analysis demonstrated a statistically significant difference among the groups (χ^2^(3) = 16.01, *p* = 0.001). Compared with the control group, DOX administration significantly reduced hepatic SIRT1 levels (*p* = 0.002). Both melatonin and agomelatine treatments significantly increased SIRT1 levels compared with the DOX group (melatonin: *p* = 0.009; agomelatine: *p* = 0.018). However, no statistically significant difference was observed between the DOX + melatonin and DOX + agomelatine groups (U = 22.0, *p* = 0.749).

### 3.4. Oxidative Stress Markers

#### 3.4.1. Effects of Different Treatments on Hepatic Antioxidant Markers

DOX administration markedly impaired hepatic antioxidant defense, as evidenced by significant reductions in Nrf2 and GSH levels (Figure 4 and Table 2). One-way ANOVA demonstrated significant overall group effects for Nrf2 (F(3,24) = 24.97, *p* < 0.001) and GSH levels (F(3,24) = 32.75, *p* < 0.001). Compared with the control group, DOX treatment significantly reduced hepatic Nrf2 and GSH levels (*p* < 0.001 for both comparisons). Co-administration of melatonin and agomelatine significantly increased both Nrf2 and GSH levels compared with the DOX group (*p* < 0.001 for melatonin; *p* = 0.0088 for Nrf2 and *p* = 0.0011 for GSH with agomelatine). However, no statistically significant difference was observed between the DOX + melatonin and DOX + agomelatine groups for either Nrf2 (*p* = 0.5441) or GSH levels (*p* = 0.7865).

#### 3.4.2. Effects of Different Treatments on Hepatic Lipid Peroxidation

As shown in Figure 4 and Table 2, hepatic MDA levels differed significantly among the experimental groups. Since the MDA data did not meet the normality assumption, nonparametric tests were used. Kruskal–Wallis analysis revealed a statistically significant difference among the experimental groups (χ^2^(3) = 20.91, *p* < 0.001). Compared with the control group, DOX administration significantly increased hepatic MDA levels (*p* = 0.002), indicating enhanced lipid peroxidation. Both melatonin and agomelatine treatments significantly reduced MDA levels compared with the DOX group (*p* = 0.002 for both comparisons). However, no statistically significant difference was observed between the DOX + melatonin and DOX + agomelatine groups (*p* = 0.338).

### 3.5. Effects of Different Treatments on Liver Inflammatory Cytokines

**Figure 5:**
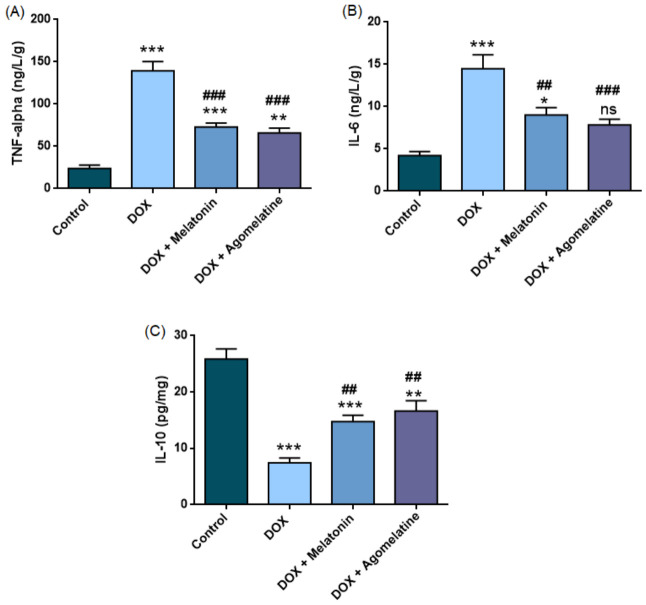
Effects of melatonin and agomelatine on DOX-induced inflammatory cytokine levels in liver tissue: (**A**) TNF-α, (**B**) IL-6, and (**C**) IL-10 levels in liver tissue of experimental groups. Doxorubicin (DOX) administration significantly increased the pro-inflammatory cytokines TNF-α and IL-6, while significantly reducing the anti-inflammatory cytokine IL-10 compared with the control group. Treatment with melatonin or agomelatine significantly attenuated the DOX-induced increase in TNF-α and IL-6 levels and restored IL-10 levels. Values are expressed as mean ± SEM (n = 7 per group). Statistical analysis was performed using one-way ANOVA followed by Tukey’s multiple-comparison test. *** *p* < 0.001, ** *p* < 0.01, * *p* < 0.05 vs. Control; ### *p* < 0.001, ## *p* < 0.01 vs. DOX; ns: not significant.

Figure 5 and Table 3 show the concentrations of hepatic inflammatory cytokines across the experimental groups. DOX administration resulted in a marked inflammatory response in liver tissue. One-way ANOVA demonstrated a significant overall group effect for both TNF-α (F(3,24) = 46.85, *p* < 0.001) and IL-6 levels (F(3,24) = 17.45, *p* < 0.001). Compared with the control group, DOX treatment significantly increased hepatic TNF-α and IL-6 levels (*p* < 0.001 for both comparisons). Co-administration of melatonin significantly reduced TNF-α (*p* < 0.001) and IL-6 levels (*p* = 0.0045) compared with the DOX group. Similarly, agomelatine treatment also significantly attenuated TNF-α and IL-6 levels (all *p* < 0.001) relative to DOX alone. However, no statistically significant difference was observed between the DOX + melatonin and DOX + agomelatine groups for either TNF-α (*p* = 0.8971) or IL-6 (*p* = 0.8456), suggesting that both treatments exert comparable anti-inflammatory effects.

In contrast to the pro-inflammatory cytokines, the anti-inflammatory cytokine IL-10 showed the opposite trend. One-way ANOVA revealed a significant overall group effect (F(3,24) = 25.96, *p* < 0.001). IL-10 levels were significantly lower in the DOX group than in the control group (*p* < 0.001). Both melatonin and agomelatine treatments significantly increased IL-10 levels compared with DOX alone (*p* = 0.0100 and *p* = 0.0011, respectively). However, IL-10 levels did not differ significantly between the DOX + melatonin and DOX + agomelatine groups (*p* = 0.8086).

Overall, these findings indicate that DOX induces a pronounced inflammatory response characterized by increased TNF-α and IL-6 levels and reduced IL-10 levels in liver tissue. In contrast, both melatonin and agomelatine treatments significantly attenuate this inflammatory response.

## 4. Discussion

Doxorubicin (DOX) is an effective anthracycline widely used in cancer therapy, but its clinical use is limited by dose-dependent toxicities, including hepatotoxicity [6,15]. Consistent with previous studies reporting hepatocellular degeneration and necrosis after DOX exposure, our study demonstrated significant hepatic injury characterized by increased hepatic 99mTc-PYP uptake and elevated AST, ALT, and LDH levels [7,8]. These findings indicate substantial hepatocellular membrane damage and necrotic injury [6,14]. Co-administration of melatonin or agomelatine significantly attenuated these alterations, suggesting that melatonergic interventions mitigate DOX-induced hepatocellular injury [16,25].

An important aspect of this study is the combined use of scintigraphic imaging and biochemical markers to evaluate liver injury [26]. Technetium-99m pyrophosphate (99mTc-PYP) has a strong affinity for calcium deposits that accumulate in necrotic tissues [26,28]. Previous reports have shown hepatic tracer localization in cases of massive hepatic necrosis [27]. DOX-induced mitochondrial dysfunction and membrane damage may disturb calcium homeostasis and promote calcium–phosphate complex formation during cell death [16]. These complexes serve as binding sites for pyrophosphate tracers, accounting for the increased tracer uptake observed in our model [26]. The parallel elevation of hepatic 99mTc-PYP uptake and serum enzymes, therefore, supports a necrosis-associated mechanism of DOX hepatotoxicity. Both MEL and AGO markedly reduced tracer accumulation, indicating protection against hepatocellular necrotic injury [19,25].

Oxidative stress is widely recognized as a central mechanism in DOX-induced liver injury [6]. Excessive production of reactive oxygen species (ROS) promotes lipid peroxidation, mitochondrial dysfunction, and depletion of endogenous antioxidant defenses [15,30]. Consistent with this mechanism, DOX treatment in the present study significantly increased MDA levels while decreasing GSH and Nrf2, indicating a shift toward a pro-oxidant hepatic environment [7,8]. Nrf2 is a key transcription factor regulating antioxidant and detoxifying genes, including those involved in glutathione metabolism [10]. Therefore, suppression of Nrf2 increases susceptibility to oxidative injury and facilitates inflammatory signaling.

Both melatonin and agomelatine significantly improved the oxidant–antioxidant balance in our model. Treatment with these agents reduced lipid peroxidation and restored GSH and Nrf2 levels compared with DOX alone. These findings are consistent with the well-established antioxidant properties of melatonin, including direct free-radical scavenging, mitochondrial protection, and stimulation of endogenous antioxidant systems [11,17]. Previous experimental studies have similarly demonstrated that melatonin attenuates DOX-induced hepatic oxidative injury and normalizes lipid peroxidation markers (6,19).

Doxorubicin-induced liver injury is not restricted to necrosis but also includes a substantial apoptotic component characterized by caspase activation, Bax/Bcl-2 imbalance, and DNA fragmentation detected by TUNEL staining [8,9,12]. In the present study, increased 99mTc-PYP uptake together with elevated AST, ALT, and LDH levels provided strong evidence of necrotic hepatocellular injury. Nevertheless, the marked improvement in oxidative stress and inflammatory parameters following melatonin and agomelatine treatment raises the possibility that apoptotic pathways may also have been attenuated. Previous studies have reported that reductions in oxidative stress and inflammatory signaling are frequently accompanied by decreased caspase activation and suppression of mitochondrial pro-apoptotic pathways in doxorubicin-induced tissue injury [8,9,10]. However, apoptotic markers were not directly evaluated in the present study, and therefore, the contribution of apoptosis to the observed hepatoprotective effects remains to be determined. Additional investigations incorporating TUNEL staining and analyses of apoptosis-related proteins would provide a more comprehensive characterization of the cell death mechanisms involved [8,9,12].

Inflammatory signaling represents another important component of DOX-induced liver injury. Oxidative stress and inflammation are closely interconnected, with ROS promoting the activation of transcription factors such as NF-κB, which drives cytokine production [10,32]. In the present study, DOX significantly increased hepatic TNF-α and IL-6 levels while reducing the anti-inflammatory cytokine IL-10, indicating a pronounced pro-inflammatory shift [9,30]. TNF-α contributes to hepatocellular injury through mitochondrial dysfunction and death-receptor signaling, whereas IL-6 participates in acute inflammatory responses and hepatocyte stress signaling [33,34]. Conversely, IL-10 suppresses excessive cytokine production and limits inflammatory amplification [35,36].

Treatment with MEL and AGO significantly reduced TNF-α and IL-6 levels and partially restored IL-10, indicating that melatonergic interventions can rebalance inflammatory signaling in DOX-induced liver injury [19,25]. Restoration of antioxidant defenses, particularly through Nrf2 activation, may contribute to this anti-inflammatory effect by suppressing NF-κB-mediated cytokine production. By limiting inflammatory amplification, MEL and AGO may reduce secondary hepatocellular injury and prevent progression of necrotic damage [6,15].

Another important mechanistic observation in this study is the involvement of SIRT1, a NAD^+^-dependent deacetylase that regulates mitochondrial function, oxidative stress responses, and inflammatory signaling [14]. Increasing evidence suggests that the SIRT1–Nrf2–NF-κB signaling axis plays a central role in controlling oxidative stress and inflammation in toxic organ injury [10,15]. In our study, DOX markedly reduced hepatic SIRT1 levels, whereas both MEL and AGO restored SIRT1 expression. This recovery occurred concurrently with improvements in oxidative stress markers, Nrf2 activity, and cytokine balance, suggesting that MEL and AGO may exert hepatoprotective effects by restoring SIRT1-mediated cellular defense mechanisms [7,14].

The concurrent suppression of SIRT1 and Nrf2 by doxorubicin and their restoration following melatonin or agomelatine treatment suggest a potential biological association between these pathways. However, the present dataset is insufficient to establish a direct causal relationship because Nrf2 activation can be regulated not only through SIRT1 but also through Keap1 redox sensitivity, PI3K/Akt/GSK-3β, AMPK, and other stress-responsive signaling pathways [7,10,15]. Although previous studies have suggested that SIRT1 may stabilize Nrf2 through deacetylation and facilitate nuclear antioxidant responses, direct confirmation of this mechanism would require additional experiments, including pharmacological inhibition, gene-silencing approaches, nuclear translocation analyses, and co-immunoprecipitation studies [10,14]. Therefore, the most appropriate interpretation of the present findings is that melatonin and agomelatine concurrently improved SIRT1–Nrf2-associated protective networks, whereas the observed recovery does not demonstrate that SIRT1 is the sole or obligatory determinant of Nrf2 activation [7,10,15].

Preclinical evidence suggests that agomelatine may exert antioxidant and anti-apoptotic effects in hepatic tissue despite the need for liver enzyme monitoring during clinical use [25]. As an MT1/MT2 receptor agonist and 5-HT2C antagonist, agomelatine may influence circadian and mitochondrial regulatory pathways that converge on cellular redox homeostasis [37]. Thus, the improvements in oxidative stress parameters observed in the MEL and AGO groups provide a plausible explanation for the reduced hepatocellular injury observed in this study [15,19].

Although agomelatine appeared hepatoprotective in the present experimental model, its requirement for liver function monitoring during clinical use may initially seem contradictory. However, this apparent discrepancy likely reflects fundamental differences in the biology of the underlying toxicities [23,24]. Clinically reported agomelatine-associated hepatic events are generally rare and idiosyncratic and characterized by time-dependent elevations of serum transaminases, whereas the doxorubicin model used in the present study represents an acute/subacute form of injury characterized by high oxidative burden and inflammatory activation, resembling an intrinsic (Type A) pattern of hepatotoxicity [23,24]. Consequently, although agomelatine may induce transaminase elevations in susceptible individuals during chronic clinical exposure, it may exert protective effects in acute oxidative–inflammatory liver injury through MT1/MT2 receptor agonism, circadian–metabolic regulation, and redox modulation. These observations suggest that the clinical and experimental findings are not necessarily mutually exclusive but may instead represent context-dependent biological responses [20,23,25]. Furthermore, the present findings should not be interpreted as evidence of unrestricted hepatic safety but rather as support for a potential protective effect under specific pathophysiological conditions [24,25].

Our findings may also have potential clinical implications. Anxiety and depressive symptoms are common in oncology patients and may compromise treatment adherence [21,22,38]. As a melatonergic antidepressant used in the treatment of major depressive disorder, agomelatine may provide dual supportive benefits by improving psychological well-being while attenuating doxorubicin-induced hepatic injury through modulation of oxidative and inflammatory pathways.

Whether melatonin interferes with the primary antitumor activity of doxorubicin remains one of the most important translational questions in the development of hepatoprotective strategies. Current evidence generally suggests that melatonin does not diminish chemotherapeutic efficacy and may, under certain circumstances, enhance treatment responses [11,17]. Because melatonin can influence oxidative stress, mitochondrial permeability, apoptotic signaling, and circadian regulation in a context-dependent manner, it has been proposed to act as a cytoprotective agent in normal tissues while potentially increasing tumor cell sensitivity to doxorubicin [11,17]. In contrast, direct evidence regarding the combined use of agomelatine and doxorubicin in tumor-bearing models remains limited [20,25]. Accordingly, the present study cannot determine whether hepatoprotection and antitumor efficacy can be preserved simultaneously during agomelatine treatment.

### Limitations

The present study has several limitations. First, although hepatic injury was consistently demonstrated by 99mTc-PYP scintigraphy, serum liver enzymes, oxidative stress markers, and inflammatory cytokines, H&E histopathology and immunohistochemical confirmation were not performed, limiting direct morphological correlation [5,26]. Second, apoptosis-related markers such as caspase-3, Bax, Bcl-2, and TUNEL staining were not evaluated; therefore, the relative contributions of necrotic and apoptotic cell death could not be fully distinguished [8,9,12]. Third, mechanistic experiments, including EX-527 administration, gene silencing, nuclear translocation analysis, and co-immunoprecipitation, were not performed; thus, the relationship between SIRT1 and Nrf2 should be interpreted as associative rather than causal [10,14,15]. Finally, the study was conducted in an acute/subacute non-tumor-bearing model and therefore cannot determine whether hepatoprotection can be achieved without compromising the antitumor efficacy of doxorubicin or whether similar effects would be maintained during long-term chemotherapy protocols [2,4,11]. Future studies incorporating histopathological validation, apoptosis-related markers, mechanistic pathway analyses, and tumor-bearing long-term models are warranted to further clarify the protective role of melatonin and agomelatine in doxorubicin-induced hepatotoxicity.

## 5. Conclusions

DOX induced significant hepatic injury characterized by increased necrosis-associated tracer uptake, oxidative stress, inflammatory cytokine activation, and reductions in hepatic SIRT1 and Nrf2 levels. Both melatonin and agomelatine significantly attenuated these pathological changes, suggesting that melatonergic modulation may protect against DOX-induced hepatotoxicity by restoring redox balance and attenuating inflammatory responses. These findings support the potential utility of melatonergic agents as adjunctive strategies for reducing doxorubicin-associated liver injury and highlight the value of 99mTc-PYP scintigraphy as a functional biomarker of hepatocellular damage.

## Figures and Tables

**Table 1:** Effects of treatments on hepatic injury markers (mean ± SEM).

Groups	Control	DOX	DOX + Melatonin	DOX + Agomelatine
Scintigraphy (99mTc-PYP uptake)	39,254 ± 3141	273,955 ± 26,387 ***	129,034 ± 12,080 ***,###	136,216 ± 5901 **,###
AST (U/L)	197.89 ± 15.12	652.13 ± 79.87 ***	428.26 ± 32.85 *,#	419.59 ± 34.57 *,##
ALT (U/L)	69.82 ± 4.51	366.00 ± 43.00 ***	195.60 ± 21.60 *,##	205.53 ± 30.39 *,##
LDH (U/L)	384.14 ± 11.49	1242.70 ± 69.08 ***	660.09 ± 69.59 **,###	746.16 ± 40.66 ***,###

Values are expressed as mean ± SEM. *** *p* < 0.001, ** *p* < 0.01, * *p* < 0.05 vs. Control; ### *p* < 0.001, ## *p* < 0.01, # *p* < 0.05 vs. DOX.

**Table 2:** Effects of treatments on hepatic oxidative stress markers (mean ± SEM).

Groups	Control	DOX	DOX + Melatonin	DOX + Agomelatine
SIRT-1	3.79 ± 0.29	1.59 ± 0.19 **	2.68 ± 0.19 *,##	2.58 ± 0.18 *,#
Nrf2	260.89 ± 22.08	92.72 ± 8.15 ***	188.47 ± 8.13 **,###	162.00 ± 12.40 ***,##
GSH	22.27 ± 1.45	6.37 ± 0.67 ***	14.95 ± 1.27 ***,###	13.44 ± 1.02 ***,##
MDA	1.65 ± 0.21	7.08 ± 0.51 **	3.35 ± 0.35 *,##	3.78 ± 0.37 **,##

Values are expressed as mean ± SEM (n = 7 per group). * *p* < 0.05, ** *p* < 0.01, *** *p* < 0.001 vs. Control; # *p* < 0.05, ## *p* < 0.01, ### *p* < 0.001 vs. DOX.

**Table 3:** Effects of treatments on inflammatory cytokines (mean ± SEM).

Groups	Control	DOX	DOX + Melatonin	DOX + Agomelatine
TNF-α	23.90 ± 3.99	139.24 ± 11.15 ***	72.78 ± 4.76 ***,###	65.91 ± 5.61 **,###
IL-6	4.20 ± 0.49	14.49 ± 1.64 ***	9.01 ± 0.87 *,##	7.83 ± 0.68 ###
IL-10	25.84 ± 1.82	7.43 ± 0.88 ***	14.73 ± 1.17 ***,##	16.61 ± 1.84 **,##

Values are expressed as mean ± SEM (n = 7 per group). *** *p* < 0.001, ** *p* < 0.01, * *p* < 0.05 vs. Control; ### *p* < 0.001, ## *p* < 0.01 vs DOX.

## Data Availability

The data that support the findings of this study are not publicly available due to ethical reasons, but are available from the corresponding author upon request. The data are not publicly available due to privacy/ethical reasons.
